# Ice Nucleation Abilities and Chemical Characteristics of Laboratory-Generated and Aged Biomass Burning Aerosols

**DOI:** 10.1021/acs.est.4c04941

**Published:** 2025-02-02

**Authors:** Jie Chen, Fähndrich Martin Othmar Jakob, Aristeidis Voliotis, Huihui Wu, Sara Aisyah Syafira, Osayomwanbor Oghama, Nadia Shardt, Nicolas Fauré, Xiangrui Kong, Gordon Mcfiggans, Zamin A. Kanji

**Affiliations:** † Institute for Atmospheric and Climate Science, ETH Zürich, Zurich 8092, Switzerland; ‡ Centre for Atmospheric Science, Department of Earth and Environmental Sciences, School of Natural Sciences, University of Manchester, Manchester M13 9PL, U.K.; § Department of Chemistry and Molecular Biology, University of Gothenburg, SE-413 90 Gothenburg, Sweden; ∥ National Centre for Atmospheric Science, The University of Manchester, Manchester M13 9PL, U.K.

**Keywords:** biomass burning aerosol, ice nucleation, photochemical aging

## Abstract

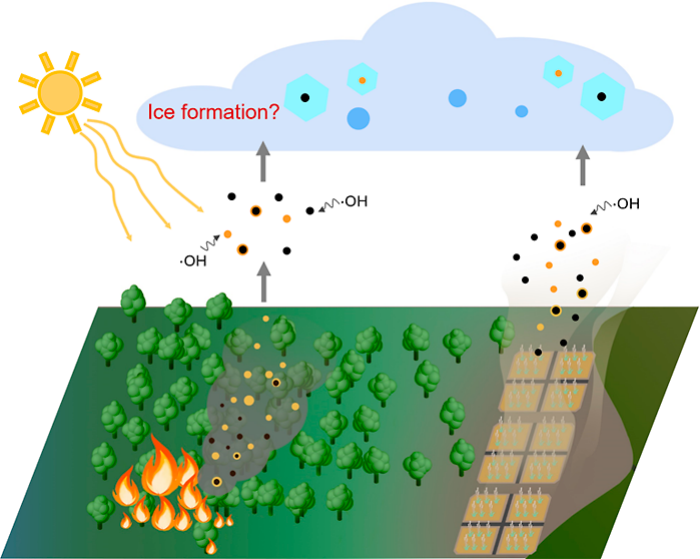

Biomass burning aerosol (BBA) contributes significantly to the global aerosol burden, yet its chemical nature and ice nucleation activities (INAs) are unconstrained due to the heterogeneity in biomass sources and complex evolution of atmospheric aging processes. This study comprehensively investigates the chemical composition and INA of BBA generated through laboratory-controlled burns with different biomasses and burning conditions. Both freshly emitted and photochemically aged BBA produced from different processes exhibit distinct and reproducible chemical compositions. However, the INA of BBA shows substantial variability at mixed-phase cloud temperatures and cannot be predicted by the chemical variability of the enriched carbonaceous materials. This indicates the negligible role of carbonaceous materials in determining the INA of BBA. Using laboratory data, we further evaluate the impact of BBA on atmospheric ice nucleation using particulate matter mass concentration and particle equivalent spherical radius. The estimated ice nucleating particle (INP) concentrations contributed by laboratory-produced BBA are lower than those observed during BBA pollution in field studies. This discrepancy is likely attributed to co-lofted mineral particles during real-world biomass burning, such as ash or soil particles, rather than carbonaceous-rich particles from combustion. We encourage further research to quantify the contribution of mineral particles to the INP concentrations of BBA.

## Abstract 2

This study explores the physicochemical nature and ice nucleation activities of freshly emitted and photochemically aged biomass burning aerosol, thereby providing insights into its role in aerosol–cloud interactions.

## 1 Introduction

Biomass burning (BB) resulting from global forest fires and domestic heating emits substantial quantities of particles^1^ and gas pollutants^2^ into the atmosphere, significantly perturbing the atmosphere-Earth climate system.^3^ In addition to their direct radiative effects through absorbing and reflecting solar radiation, biomass-burning aerosols (BBA) can alter cloud microphysics and optical properties by serving as cloud condensation nuclei (CCN)^4,5^ and ice nucleating particles (INPs),^6−9^ thereby causing indirect radiative effects.^10^ However, the inconclusive ice nucleation activities (INAs) of BBA derived from various biomass sources and the lack of a representative ice nucleation parametrization for BBA have led to poorly constrained aerosol-ice cloud interactions caused by BBA.

Previous laboratory and field studies have reported highly variable INP concentrations originating from BBA.^6,7,11^ Some of these studies found detectable INPs in BB plumes, but INAs of the generated BBA exhibit variations among different studies.^7,12^ Conversely, a few studies suggested that BBA had a negligible contribution to INPs relevant for mixed-phase clouds.^11,13^ The contradicting results are likely caused by the diverse chemical compositions and mixing state of BBA, attributed to fuel type, combustion conditions, meteorological conditions during the aerosolization and the atmospheric aging processes of BBA. Currently, it remains unclear which of the primary BBA components, such as black carbon (BC), organic matter (OM), or inorganic components/minerals, would contribute to the INA of BBA. BC particles have been proven to have negligible INAs under mixed-phase cloud conditions (−38 to 0 °C). Schill et al.^9^ suggest that BC explains only ∼4% of BBA INPs at mixed-phase cloud temperatures, while organic INPs are ten times more abundant in fresh BBA compared to BC INPs. The importance of ash particles as INPs in BBA has been suggested by previous studies,^8,14,15^ is and is likely associated with the ice-active mineral phase.^16^ However, the prevalence of mineral-containing particles in BBA and their contribution to the atmospheric INP population of BBA has yet to be quantified.

BBA undergoes complex atmospheric aging processes after its emission. The role of atmospheric aging on the INA of BBA has been studied and yields conflicting conclusions. One early study indicated that photochemical aging does not influence the INA of wood-burning aerosols, due to the absence of uniform organic coating on the particle surface formed from the gas phase.^17^ In contrast, Jahl et al.^18^ observed enhanced INAs of BBA from grass fuels after being photochemically aged by hydroxyl (OH) radicals. This change was explained by the removal of organic coatings that conceal the mineral-based ice-active sites through oxidation during the aging process. The divergent findings of these studies hint that the transformation of organic compounds during photooxidation probably exerts a significant influence in regulating the INAs of BBA.

The unresolved INAs of BBA are likely linked to the inherent chemical properties of BBA and the evolution of organic compounds during aging. Precise chemical characterization of BBA alongside the INA measurements is required to address the gap and elucidate the underlying mechanism. In this study, BBA was generated from different fuels through controlled laboratory burning experiments and subjected to atmospheric-relevant oxidation conditions for aging. The INAs of fresh and photochemically aged BBA were quantified under mixed-phase cloud conditions. Simultaneously, we conducted comprehensive chemical characterizations of BBA using a combination of online and offline techniques. These methods cover analyses of BBA bulk-phase and single-particle compositions, with a specific focus on organic compounds.

## 2 Methodology

### 2.1 Biomass Burning Chamber Experiments

**Figure 1:**
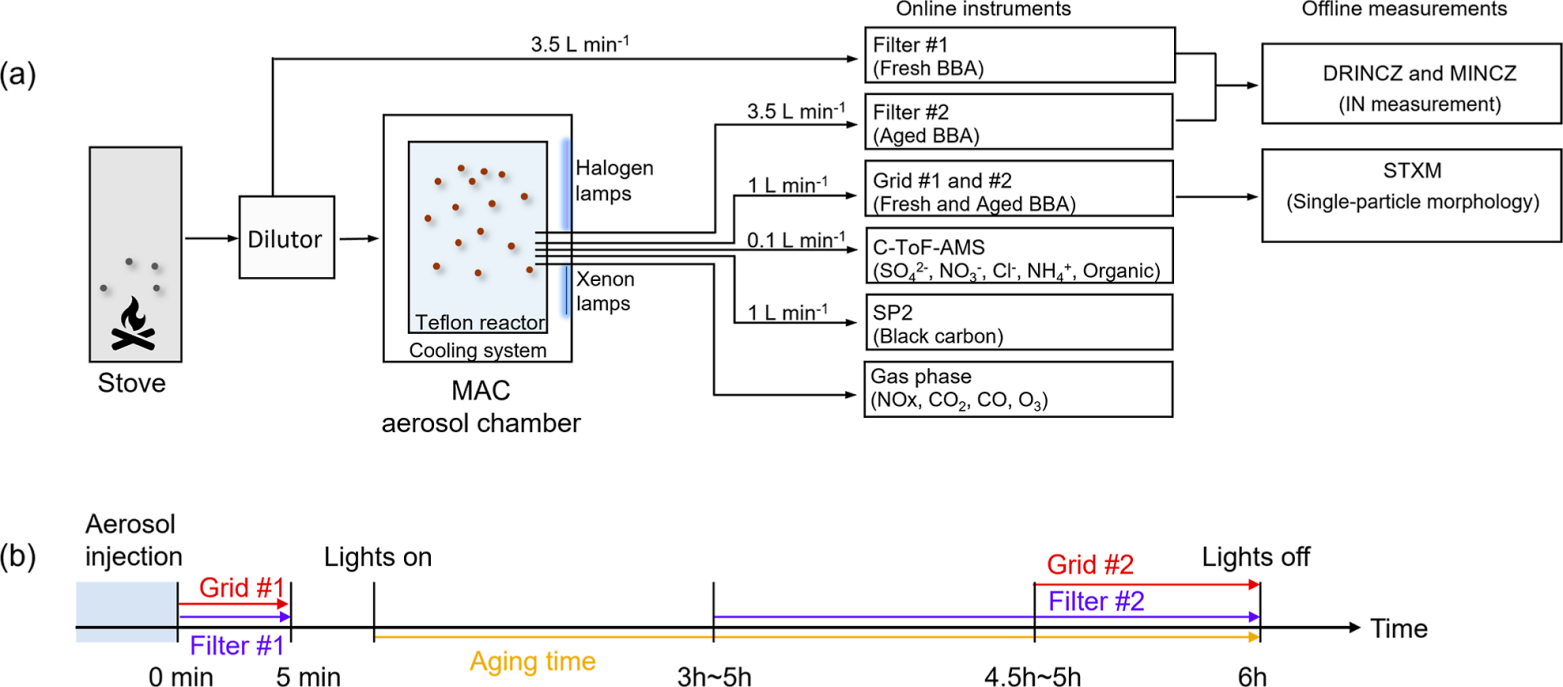
Schematic of the experimental setup of (a) biomass burning chamber experiments and (b) the time scale for filter- and grid- collection. Fresh BBA and single BB particles were collected onto filter #1 and grid #1, while aged BBA and single BB particles were collected onto filter #2 and grid #2, respectively. The starting time for the collection of grid #1 and filter #1 is designated as 0 min (b), corresponding to the end of the aerosol injection. Abbreviations: MAC: Manchester aerosol chamber; C-ToF-AMS: the compact time-of-flight aerosol mass spectrometer; SP2: single particle soot photometer.

The instrument configuration for the biomass burning chamber experiment is shown in Figure 1a. Different types of biomasses were burned to generate aerosol. The selected biomasses include hardwood (beech), softwood (ponderosa pine), leaves (hornbeam), and peat, which are representative biomass types for wildfire and residential heating.^6,19,20^ Biomass was ignited and burned in a stove through either flaming combustion or smoldering combustion (see more details at Text S1 about the combustion type control). The freshly emitted BBA was diluted (DEKATI dilutor) and injected into the Manchester Aerosol Chamber (MAC, see more details in Shao et al.^21^). The injection process took 15–30 min until particle mass concentration reached 179.1 ± 50.2 μg m^–3^. The MAC chamber is a fluorinated ethylene propylene (FEP) Teflon chamber and has a volume of 18 m^3^. The temperature and relative humidity (RH) within the chamber can be controlled over a wide range, enabling the study of aerosol under atmospheric-relevant conditions. In this study, the chamber experiments were conducted at 25 ± 1 °C and at 50–60% RH. The chamber is equipped with a set of halogen bulbs and two arc xenon lamps, collectively providing an atmospherically relevant actinic flux spectrum.^21^ When the injected BBA is exposed to these radiation sources, it undergoes photochemical aging. The typical concentration of OH within the chamber is 0.8–1 × 10^6^ molecules cm^–3^, creating atmospherically relevant oxidative conditions.^22^ No additional oxidants were introduced into the chamber during aging experiments. Before each experiment, the chamber was filled and flushed with a high flow of purified air (3 m^3^ min^–1^) for 2 h to establish a low background condition. While harsh cleaning procedure was conducted weekly (see more details at Text S2).

Both the fresh and aged BB particles were sampled on 47 mm (diameter) polycarbonate filters and TEM grids (Pella, Inc., 01754-F). These filters and grids were later used for the offline ice nucleation measurements of bulk BBA and chemical morphology analysis of individual particles, respectively. In each experiment, filter samples of fresh and aged BBA were collected using two customized aerosol samplers (with 47 mm in-line filter holders, Pall Lab) at a flow rate of 3.5 L min^–1^. Fresh BBA was collected directly from the stove onto filters for 5 min (Figure 1a), taking advantage of the sufficiently high aerosol concentration. The collection of aged BBA begins after a few hours of radiation exposure. The total sampling time for aged BBA was determined by the concentration of aerosol particles remaining within the chamber. The exact sampling time points for each experiment can be found in Table S2. Sampling was stopped if the particle concentration was too low due to wall losses or if the signal-to-noise ratio for measuring black carbon (BC) by the SP2 (single particle soot photometer, Droplet Measurement Technologies) was too low. The timeline for filter and grid sampling is schematically shown in Figure 1b. Grid samples were collected at a rate of 1 L min^–1^ by a DKL-2 sampler (Genstar Electronic Technology, China) from the MAC chamber. Fresh and aged BBA was collected onto a grid for 5 min and 1 h, respectively. It is important to note that aerosol within the chamber underwent continuous aging while sampling, as indicated by Figure 1b. Consequently, the aging time of collected particles on one filter or grid can be different. All characterizations of aged BBA using sample filters or grids reflect an average representation of aged particles (see more details in Section 2.3). Sample filters were transported and stored at −20 °C until analysis. Information about the BBA samples, including the biomass types, burning conditions, sampling and aging time, is detailed in Table S1.

### 2.2 Ice Nucleation Measurements at Mixed-phase Cloud Temperature

The ice nucleation of BBA via immersion freezing was measured by the droplet ice nuclei counter Zurich (DRINCZ, David et al.^23^) and microfluidics ice nuclei counter Zurich (MINCZ, Isenrich et al.^24^) at mixed-phase cloud temperatures (from −38 to 0 °C). DRINCZ and MINCZ are both custom-designed instruments built following the working principle of the droplet freezing technique. DRINCZ measures droplets in sizes of the microliter range (50 μL) whereas MINCZ allows the detection of picoliter-size droplets (∼200 pL). Droplets of different sizes freeze within specific temperature ranges, enabling the examination of INP number concentrations at different temperature conditions. Thereby, DRINCZ and MINCZ measure the INPs activating at temperatures ranging from −25 to 0 °C and −38 to 0 °C, respectively.

The sample filters were immersed in 7 mL ultrahigh purity water (Sigma-Aldrich, W4501-1L) and shaken by the ultrasonic bath for 30 min to form BBA suspensions. The volume of ultrahigh purity water was selected based on the minimum sample volume required for DRINCZ (∼5 mL) and MINCZ (less than 1 mL) measurements, amounting to a total of 7 mL, with an additional 1 mL reserved. The minimum water volume ensures the highest possible particle concentration in the resulting sample suspensions and maximizes the likelihood of detecting the rare INPs within them. To avoid the evaporation of volatile components in samples, ice was added to the ultrasonic bath to maintain a low bathtemperature. Microliter-size droplets (50 μL, ∼4.5 mm in diameter if a spherical shape was assumed) were pipetted from the sample suspensions and placed into a 96-well polypropylene tray (732–2386, VWR, USA). Picoliter-size droplets with a diameter of 75 μm were produced by a microfluidic chip (described in Isenrich et al.^24^ and Shardt et al.^25^) from the same suspension and stored in PFA tubing (50 cm in length, 360 μm o.d., 75 μm i.d., IDEX Health & Science LLC, USA). The 96-well tray and the PFA tubing were cooled in the ethanol bath of DRINCZ and MINCZ respectively, at a cooling rate of 1 °C min^–1^. The phase of the droplets was continuously recorded by the camera mounted on top of the ethanol bath. Customized MATLAB (DRINCZ) and Python (MINCZ) software were employed to analyze the captured images, identifying the freezing temperature of each droplet based on its optical changes upon phase transition. The frozen fraction (*f*_ice_) of the droplet population was determined by the number of frozen droplets (*N*_frozen droplets_) at a certain temperature and total droplets (*N*_total droplets_), based on eq 1
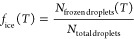
1*N*_total droplets_ in a DRINCZ experiment is 96. It can vary in MINCZ experiments due to uncertainties associated with droplet generation using microfluidic chips. *N*_total droplets_ in the present MINCZ measurement is 107 ± 27. The background of the freezing experiment was determined by measuring the freezing ability of droplets obtained by washing blank filters with ultrahigh purity water. The *f*_ice_ of droplets obtained from suspensions of blank filters (*f*_ice,BG_) indicates freezing introduced by blank filters and water impurities and is thereby used as a negative control. *f*_ice,BG_ of DRINCZ and MINCZ measurements are shown in Figure S1.

The number concentration of INPs per unit volume of sample droplet (*K*(*T*)) was calculated based on eq 2, according to Vali^26^
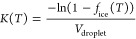
2where *V*_droplet_ is the droplet volume, which is 50 μL for DRINCZ and ∼200 pL for MINCZ experiments. Note that *K*(*T*) (mL^–1^) of filter background () needs to be subtracted from that of BBA samples (*K*(*T*)) to exclude the INPs contributed by filters or water impurities at identical temperatures (see Text S3 and Figure S1). The temperature uncertainties of DRINCZ and MINCZ are ±0.9 and ±0.2 °C respectively, including uncertainties arising from thermocouples. More details on the uncertainties of the two instruments can be found in David et al.^23^ and Isenrich et al.^24^

The INA of BBA was quantified by the active site density per unit of particle mass (*n*_m_(*T*), g^–1^), which was estimated based on INP number per sample volume (*K*(*T*) – *K*(*T*)_BG_, mL^–1^) and the particle mass concentration of sample suspension (*C*_s_, g mL^–1^), according to eq 3
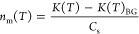
3where *C*_s_() is given by the ratio of particle mass sampled onto each filter (*M*) and the volume of water used to wash the filters (*V*_water_). *M* (g) was determined by the mass difference of each filter before and after sampling (Table S1). In our experiment, *V*_water_ is 7 mL.

The *n*_m_(*T*) obtained from our studies can be used to estimate the INP number concentration in the air (*N*_INP_(*T*), L^–1^) in biomass burning plumes using eq 4. This conversion utilizes the mass concentration of PM_2.5_ particles (*C*_PM_2.5__, μg m^–3^) measured during BBA events reported in previous field studies.
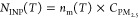
4The resulting *N*_INP_(*T*) represents the potential INP concentration that can be attributed to BBA generated from laboratory-controlled burns, assuming the same particle properties as observed in real BBA events. Comparing these *N*_INP_ values with those reported in field measurements during BBA pollution allows us to assess the atmospheric relevance of the BBA produced here.

The derived *n*_m_(*T*) in our measurements can also be used to estimate the equivalent particle radius of INPs (*r*_e_) required in one droplet to initiate immersion freezing, assuming that the most active INP initiated freezing (eq 5). This is achieved by assuming a spherical shape of BB particles with a density of 1.26 g cm^–3^, which corresponds to the mean density reported for wood-burning aerosols generated from a similar experimental setup.^27^
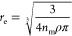
5where  is the particle volume of an INP which is equal to the volume of a spherical particle (). The equivalent INP surface area required to initiate freezing within the droplet at a given temperature (*S* = 4π*r*_e_^2^) can be determined by *r*_e_.

### 2.3 Chemical Characterizations of BBA Bulk-phase and Single Particle

The chemical compositions of BBA were characterized in real-time by instruments placed downstream of the chamber, as depicted in Figure 1a. The mass concentration of nonrefractory components in the aerosol condensed phase, including organic compounds (Org), sulfate (SO_4_^2–^), nitrate (NO_3_^–^), ammonium (NH_4_^+^) and chloride (Cl^–^) were measured by C-ToF-AMS (the compact time-of-flight aerosol mass spectrometer). The O/C ratio from C-ToF-AMS is calculated based on the empirical fit between f44 and the O/C ratio, as indicated in Aiken et al.^28^ The mass concentration of refractory BC was measured by an SP2. The laser-induced incandescence was employed to measure the refractory BC mass of individual BC particles with an intracavity Nd/YAG laser operating at 1064 nm. More details about the working principle and calibration of C-ToF-AMS and SP2 can be found in Hu et al.^27^ The measuring time for fresh and aged aerosols by C-ToF-AMS and SP2 can be found in Table S2. To maintain consistency of time resolution with the INP measurements using aerosol sample filters, the mass concentrations of chemical components for BBA discussed in the subsequent sections represent the mean values of data recorded throughout filter sampling time. Specifically, the reported chemical composition of fresh BBA is the averaged values measured from “aerosol injection” to “light on” (Table S2), while for aged BBA it represents the averaged values measured from “aged filter sample start” to “aged filter sample end” (Table S2). The gas phase within the chamber including NO_*x*_, CO, CO_2_ and O_3_ and oxygenated organic compounds was monitored by a series of analyzers and a chemical ionization mass spectrometer, but not discussed in the present study.

The chemical composition and morphology analysis of individual BB particles collected on TEM grids was conducted using synchrotron-based scanning transmission X-ray microscopy (STXM) coupled with near-edge X-ray absorption fine structure spectroscopy (NEXAFS). The STXM-NEXAFS measurements were performed at the BL4U beamline in UVSOR-III in Okazaki, Japan.^29^ Samples are subjected to soft X-rays of adjustable energy inside a vacuum chamber. At energies near the ionization threshold, inner-shell electrons of an atom can absorb photons to become excited into unoccupied orbitals, which is referred to as the absorption edge. This absorption edge is element-specific and can aid in identifying the chemical composition of particles detected. The carbon K-edge NEXAFS spectra of individual particles were detected over a photon energy range from 280 to 305 eV, which also covers the absorption of potassium L-edge at 297 eV. These carbon spectra provide information on the structure of carbon bonds and functional groups present in individual BB particles.^30^ Data processing including image alignment, correction for background signal and conversion of flux data to optical density was performed using AXIS 2000 (software developed by the Hitchcock group, http://unicorn.mcmaster.ca/aXis2000.html).

## 3 Results and Discussion

### 3.1 Chemical Properties of Fresh and Aged BBA

The chemical compositions of fresh BBA generated in each burning experiment, including Org, SO_4_^2–^, NO_3_^–^, NH_4_^+^, Cl^–^ and BC are shown in Figure S3. The mass concentrations of these components in each BB smoke are detailed in Table S3, which represent the averaged values recorded during the filter sampling time (detailed in Section 2.3). Distinctive chemical compositions have been found in fresh BBA originating from various biomass types and burning conditions (Figure S3). The lowest Org content is found in BBA generated from softwood flaming combustion (10.1% ± 0.2%) compared to those generated from smoldering combustion (90.6% ± 10.8% across different biomasses). This is expected and consistent with other studies^31,32^ that report flaming combustion produce more BC than smoldering combustion that predominantly yields Org. Table S3 shows that the peat-burning aerosol exhibits the highest Org content (98.4% ± 0.3%), followed by aerosol from the combustion of leaves (94.4% ± 2.0%), smoldering phase wood burns (75.0 ± 1.5%, mean value for softwood and hardwood) and flaming phase wood burns (10.1% ± 0.2%). The good agreement in chemical compositions of BBA generated from identical biomass type in the same burning phase during repeated experiments (Figure S3), indicates stable and reproducible chemical characteristics of the aerosol in laboratory-controlled burns. The chemical compositions of aged BBAs are shown in Figure S4. The mass fractions of different chemical components of aged BBA show a generally similar trend to those obtained from the fresh aerosol (Figure S4 versus Figure S3, Table S3). Similar to fresh BBA, aged peat-burning aerosols exhibited the highest Org content (94.7% ± 1.7%), followed by leaf- (90.9% ± 1.6%) and wood-burning aerosols (between 22.4% ± 1.5% and 78.1% ± 0.9%, including softwood and hardwood). Similar to fresh softwood-flaming aerosol, aged aerosol produced by the flaming combustion of softwood still has the highest BC loading (68.7% ± 5.4%) compared to those produced from the smoldering combustion of other fuels (between 0.4% and 15.5%). The increased Org content observed in all types of wood-burning aerosol (Table S3), as discussed in the following text, indicates the oxidation and condensation of organic compounds during photochemical aging.

**Figure 2:**
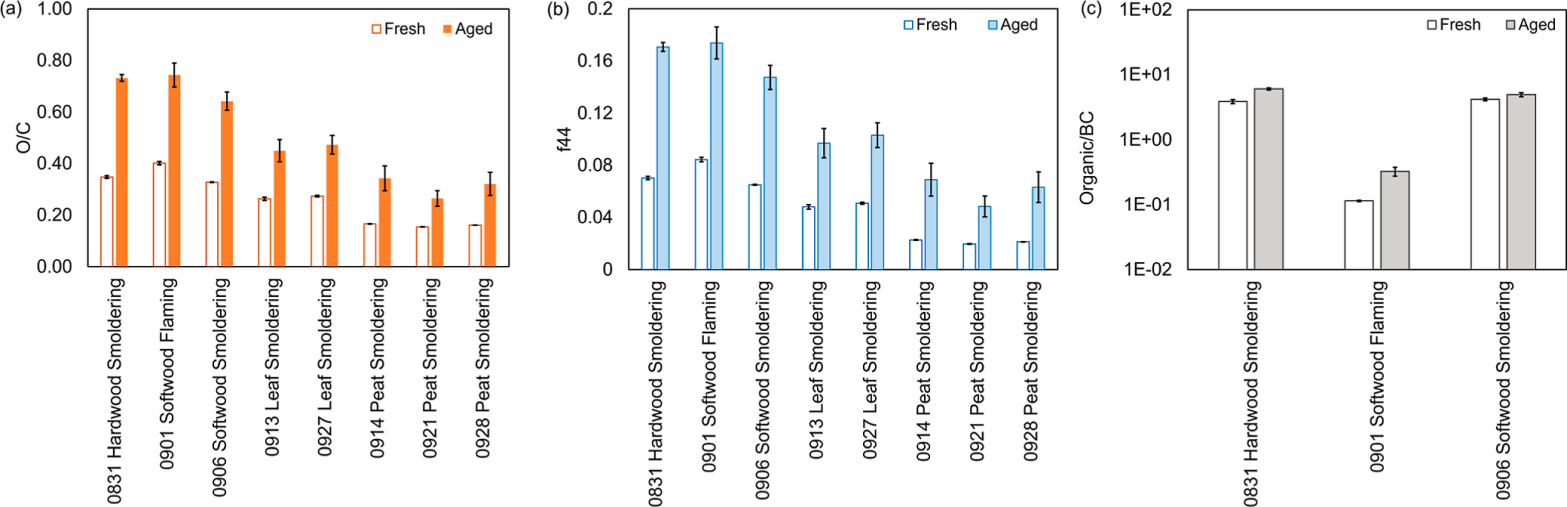
Chemical characteristics of fresh (empty bars) and aged (solid bars) BBA generated in each burning experiment. Panel (a) presents the oxygen-to-carbon ratio (O/C), (b) depicts the intensity of *m*/*z* 44 mass spectral fragment (f44) and (c) illustrates the organic-to-black carbon mass ratio (Org/BC), all obtained from C-ToF-AMS and SP2 measurements. The vertical error bars represent the standard deviations of measured characteristics by C-ToF-AMS and SP2 over the filter sampling time.

Three chemical characteristics of generated BBA, including oxygen to carbon elemental ratio (O/C), the Org to BC mass ratio (Org/BC) and the intensity of *m*/*z* 44 mass spectral fragment (CO_2_^+^ signal, referred to as f44 hereafter), are shown in Figure 2. The O/C (Figure 2a) and f44 (Figure 2b) serve as indicators of oxygenation degree of aerosols.^33^ For fresh BBA, wood-burning aerosol exhibits the highest oxygenation, as reflected in the highest values of O/C (0.36 ± 0.04) and f44 (0.07 ± 0.01), which is followed by BBAs from leaf-burning (O/C: 0.27 ± 0.01, f44: 0.05 ± 0.00) and peat-burning (O/C: 0.16 ± 0.01, f44: 0.02 ± 0.00). The O/C values of wood-burning aerosols (0.36 ± 0.04) are comparable to those reported by Fortner et al.^33^ for ponderosa combustion aerosol (0.23–0.3). The values of these two characteristics (O/C and f44) significantly increased after photochemical aging, as shown by solid bars in Figure 2a,b, indicating a higher oxygenation of aged BBA. The Org/BC of wood-burning aerosols shown in Figure 2c varies from 0.11 to 4.20. The aerosol generated from softwood flaming combustion has comparable Org/BC values (0.11 ± 0.00) with those reported by Chou et al.^17^ (0.05–0.65). This also confirms that BBA from flaming combustion produces a considerable amount of BC relative to Org, as compared to that from smoldering burning. Our findings again highlight the role of burning conditions in regulating the chemical compositions of BBA, consistent with previous studies.^31,32^ Note that the Org/BC of leaf- and peat-burning aerosols is not discussed here, given the negligible BC was produced in these aerosols (0.01–1.74 μg/m^3^ and <3.4% of the total aerosol particle mass, Table S3), falling below the limit of detection. The Org/BC of wood-burning aerosols experiences a slight increase after undergoing photochemical oxidation (Figure 2c). Note that the parameters derived here represent averaged values over the filter sampling time (Section 2.3); therefore, the change should be more significant for aerosols aged for a longer time. The increase in Org/BC can be explained by the condensation of secondary formed Org in BBA during photochemical aging, as also observed by previous studies.^34,35^ Oxidation of the organic gas phase can produce condensable species with lower volatility, leading to an increase in Org content.^34^

**Figure 3:**
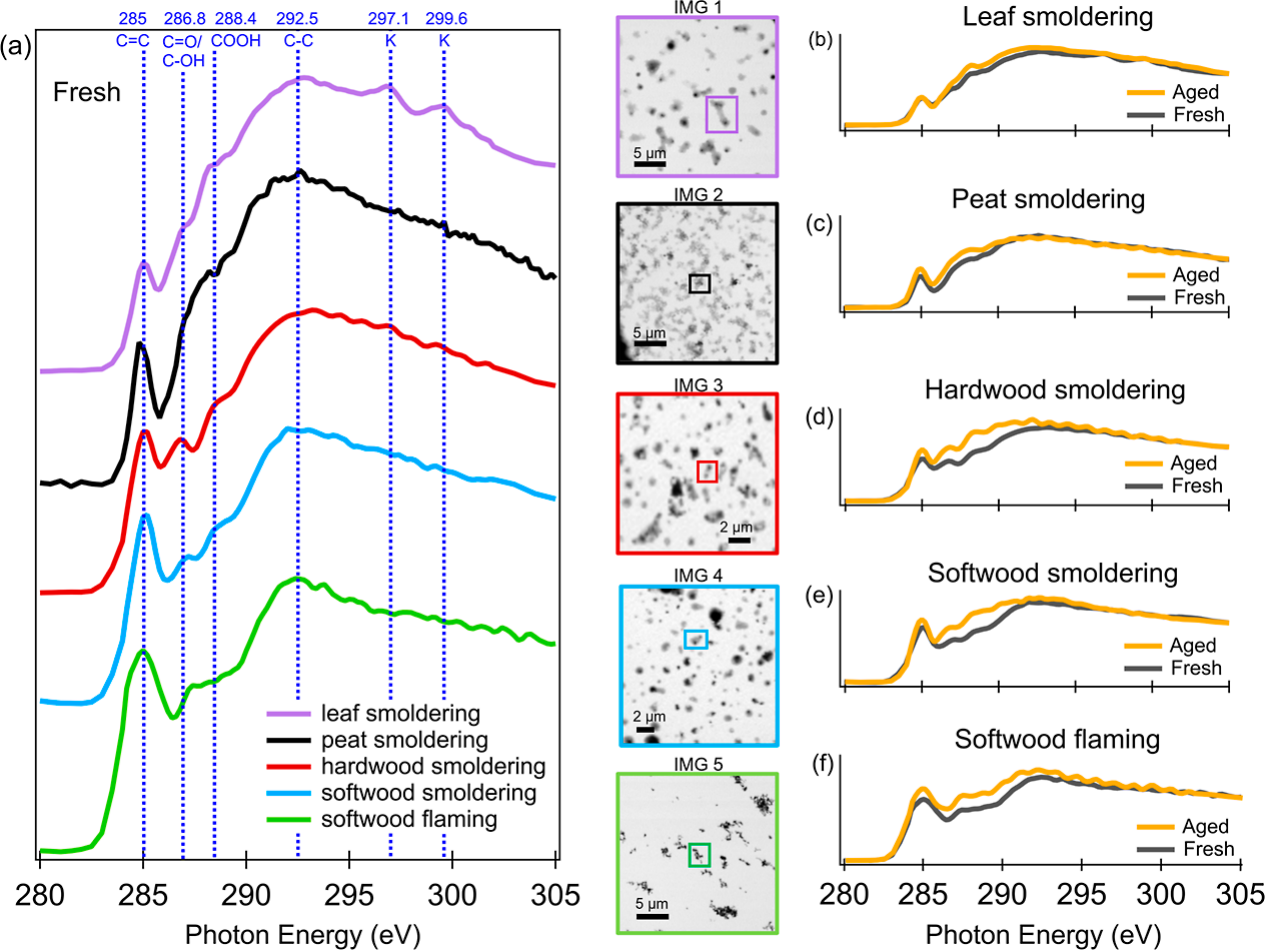
Carbon K-edge spectra of (a) fresh and (b–f) aged biomass-burning particles originating from different fuels and burning conditions. The detected particles are specified in images (IMGs in the middle panel). The morphology of fresh particles is also shown by images collected at 300 eV. (b) Particles from smoldering combustion of leaves; (c) particles from smoldering combustion of peat; (d) particles from smoldering combustion of hardwood; (e) particles from smoldering combustion of softwood; (f) particles from flaming combustion of softwood. The blue dashed lines in (a) indicate the transition energies of functional groups identified in the carbon spectra, which is also summarized in Table S4.

The chemical and morphological properties of individual BB particles were further characterized by STXM-NEXAFS. The images (IMG) of BB particles collected on the TEM grid are shown in Figure 3 (middle panel). The selected carbon K-edge spectra of individual particles, along with the identified functional groups and their corresponding transition energy ranges are illustrated in Figure 3a (also detailed in Table S4). Additional carbon spectra of individual particles are displayed in Figure S5. The repeatable shape features observed in spectra detected at different areas within each particle (Figure S5) suggest a well-mixed carbon composition of the analyzed particle and validate the representativeness of the spectrum shown in Figure 2a. While the shape of the carbon spectra varied among biomass types and burning conditions, all exhibit two prominent peaks at ∼285 eV (arising from the C 1s → π*R(C*=C)R transition) and at ∼292.5 eV (attributed to C 1s → σ*C*–C transition). These features are indicative of absorption spectra associated with BC-containing particles.^30^ Not all samples exhibited considerable BC content in the bulk aerosol measurements conducted by C-ToF-AMS (Figures S3 and S4), as the reported values represent the averaged characteristics of aerosols over the sampling time and particle population. In contrast, the results in Figure 3 obtained by STXM-NEXAFS, are based on selected individual particles. Note that particles from flaming combustion of softwood (green line in Figure 3a) exhibited distinctive spectral features compared to particles from other sources. This spectrum shows strong absorption at 285 eV with an intensity comparable to that at the post-edge energy (305 eV in the present work) attributed to total carbon. The ratio of absorbance at 285 and 305 eV (denoted as 285/305 eV) is close to 1 (0.97, Table S5), as demonstrated by previous studies,^30^ signifying its BC origin. Moreover, the absorbance peak observed at 285 eV is broader than in other cases, especially toward the lower energy end. This broadening of the peak is likely due to the presence of the C=C bond within the quinone ring structure (peaked at 284.2 eV) in the soot particles.^30^ These particles show a fractal structure (Figure 3, IMG 5), similar to freshly emitted soot particles observed by Pang et al.,^36^ which further confirms the significant presence of soot particles. Peat- and leaf-burning particles have similar carbon spectra (blue and red lines in Figure 3a), indicating their common chemical features. The 285/305 eV ratio of these particles is 0.57 and 0.69 respectively (Table S5), suggesting lower BC compared to Org. The high Org content of peat- and leaf-burning particles is also evident from their morphology (Figure 3, IMG 1 and 2), where the majority of particles appear gray on the TEM grid and differ from the dark appearance of soot particles from flaming combustion (IMG 5). Similar carbon spectra were also observed for particles resulting from the smoldering combustion of both softwood and hardwood (blue and red lines in Figure 3). Their 285/305 eV ratios (0.92 and 0.79) fall between the values of particles from flaming combustion and peat/leaf-burning, implying the intermediate chemical properties between BC-abundant (flaming combustion) and Org-enriched (peat/leaf-burning) BBA. Particles produced under such conditions displayed a core–shell structure similar to that of leaf/peat-burning particles (Figure 3, IMG 3 and 4). However, a small portion of the particles appeared gray, indicating a relatively lower organic content in wood-burning particles from smoldering combustion compared to leaf/peat-burning particles. This observation is consistent with the findings from 285/305 eV ratios and chemical characterization of bulk BBA discussed above. The spectral features in the energy range from 285 to 290 eV are associated with oxygen-containing functional groups (Table S4). Peat/leaf-burning particles have stronger absorptions in this energy range compared to wood-burning particles (Figure 3a), confirming a significant presence of Org. All these findings align with those observed for bulk BBA (Figure 2), where aerosols from the smoldering combustion of leaf and peat are primarily composed of Org, which distinguishes them from softwood-burning aerosols from flaming combustion characterized by considerable soot content (Figures 2 and S3). It is worth noting that a pronounced absorption of potassium (K) at 297.1 and 299.8 eV (transitions of K 2p_1/2_ → σ* and K 2p_3/2_ → σ*)^30^ was only observed in the leaf-burning particles, indicating the presence of detectable K in the investigated particles. The absence of K in BB particles generated from other biomass types may be attributed to differences in the inherent nature of biomass. This is consistent with the results presented by Jahn et al.,^16^ where a lower number fraction of K-containing particles is detected in wood-burning aerosol (29.7%) compared to grass-burning aerosol (55.8%–79.7%) determined using energy dispersive X-ray spectroscopy (EDX). Our results suggest that the prevalence of K can vary in BBA from different combustion sources and relying on K as a universal tracer for BB emissions may underestimate atmospheric BBA burden. For example, the contribution of BBA from wood-burning aerosol and peat-burning aerosol might be underestimated due to its lower emission of K compared to leaf-burning aerosol.

Comparing the carbon spectra of fresh and aged BB particles (Figure 3b–f), we observed an increase in absorption in energy ranges from 285.0 to 290.0 eV for all particle types after photochemical aging. This indicates the formation of oxygen-containing functional groups, either from the oxidation of existing organics in the particle phase or from gas-phase organic compounds condensing, contributes to the increased oxygenation degree of BB particles during aging. The increased absorption at 288.3 eV of the wood-burning particles after photooxidation has also been observed by Zelenay et al.^37^ This increase is consistent with the enhanced O/C and f44 observed in bulk BBA after aging (Figure 2a,b), indicating the significantly increased oxygenation of aged BBA. In addition, the increase is more pronounced in wood-burning particles (Figure 3d–f) compared to that in peat/leaf-burning particles (Figure 3a,b), aligning with that observed for the bulk BBA (Figure 2a,b). The results from both online and offline measurements, as well as bulk aerosol and single particle analysis, demonstrate the presence of distinct chemical species in BBA originating from different fuels and burning conditions. The noticeable increases in the oxygenation of BBA after aging are also evident.

### 3.2 INAs of BBA from Different Fuel Types and Impact of Photochemical Aging

**Figure 4:**
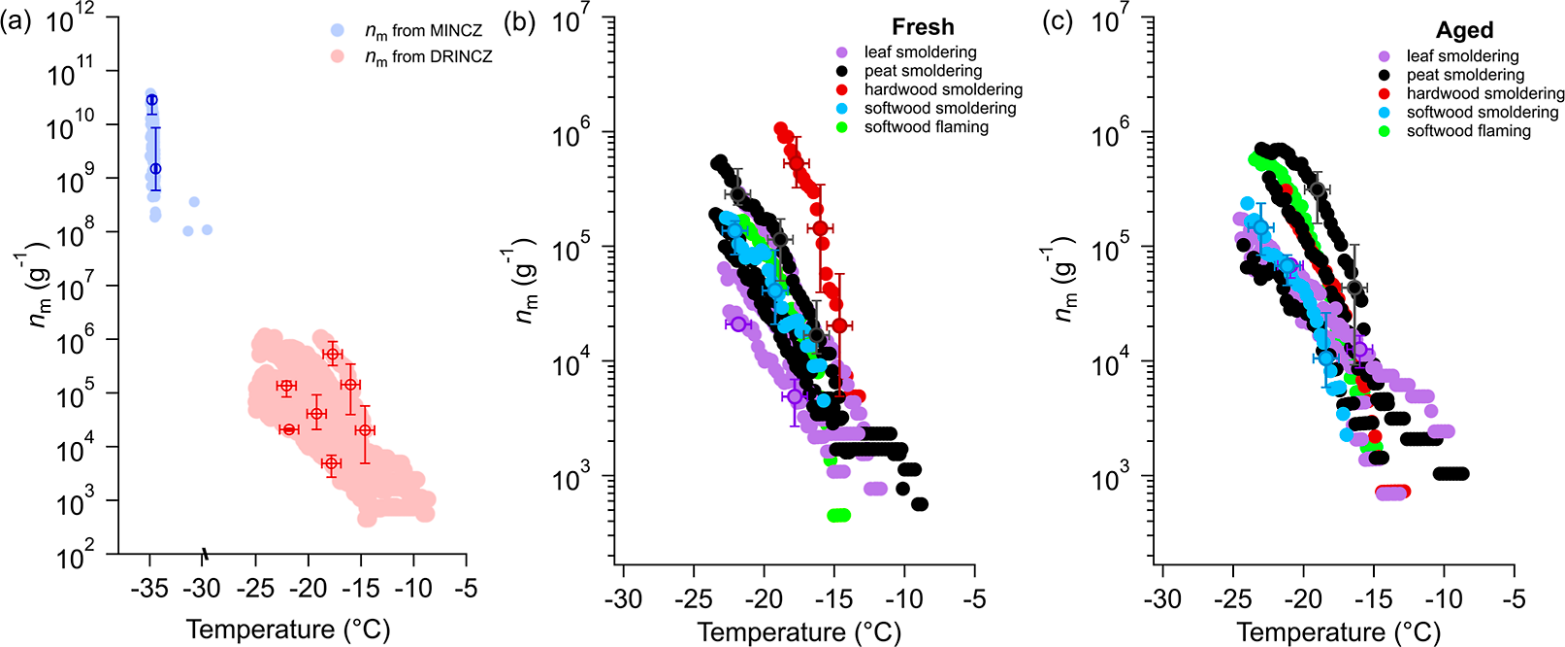
Overview of active site density per unit of biomass burning particle mass (*n*_m_) as a function of temperature derived from DRINCZ and MINCZ measurements (a); separated for (b) fresh and (c) aged BBA generated from different fuels. The horizontal error bars represent a temperature uncertainty of ±0.9 °C in DRINCZ measurements^23^ and ±0.2 °C in MINCZ measurements. The vertical error bars are deviations of *n*_m_ resulting from this temperature uncertainty. Only error bars of select data points are shown here to avoid overcrowding the figures. The data measured by MINCZ are only shown in (a), but not further discussed in (b,c).

The *f*_ice_ of droplets derived from BBA sample filters (referred to as BBA droplets hereafter) and those derived from blank filters (referred to as background droplets hereafter) is shown in Figure S1. Microliter-size BBA droplets exhibit higher freezing abilities compared to background droplets in the temperature range from −7 to −28 °C (Figure S1a), indicating that the BBA mass contained in the droplets is sufficient to nucleate ice under the observed temperature conditions. The *f*_ice_ of picoliter-size BBA droplets overlaps with that of background droplets in the temperature range from −30 to −37 °C, indicating a negligible number of INPs present in such small droplets. This is expected as smaller droplets have less BBA mass and thus lower the probability of including INPs compared to larger droplets. Given that 70%–100% of the background droplets freeze within a very narrow temperature range between −34.3 and −35 °C (Figure S1b) and a comparable mean freezing temperature have been observed for sample droplets (−34.7 °C) and background droplets (−34.8 °C, Figure S2), we consider data obtained at approximately −35 °C using MINCZ is primarily attributed to the homogeneous freezing of background droplets. The *n*_m_ of BBA is derived based on eq 3, representing the INP number that can be produced per unit mass of BBA. Only the *n*_m_ of sample droplets with higher than those of the water background (Figure S1) are shown in Figure 4a, indicating a detectable mass loading of BB INPs within a droplet to initiate its freezing. *n*_m_ obtained from MINCZ measurements present a few data points at −30 °C after excluding data points obtained at temperatures comparable to the homogeneous freezing temperature of background droplets (−34.8 °C, Figure S2). The undetectable INAs of BBA in picoliter-size droplets at temperatures below −30 °C by MINCZ is an important finding and of atmospheric relevance, as it suggests BBA produced under our conditions have a minor influence on freezing of droplets with such small size given their insufficient mass in these droplets. Cloud droplets in the real atmosphere typically range from 2 to 25 μm in radius,^38^ which is even smaller than those generated by the MINCZ system (with a radius of 37.5 μm). If the same mass concentration of BBA is assumed to be included in cloud droplets in the real atmosphere, BBAs produced here would not nucleate ice by immersion freezing.

DRINCZ exhibits higher *n*_m_ of BBA ranging from 6.3 × 10^2^ to 1.1 × 10^6^ g^–1^ within a temperature range from −8.2 to −23.2 °C. *n*_m_ derived from MINCZ measurements only present a few data points at −30 °C. As a result, only data points from DRINCZ will be included in the following discussions regarding the influence of biomass types and atmospheric aging on INAs of BBA. The *n*_m_ exhibits variability of 2 orders of magnitude at identical temperatures among different biomasses, as shown in Figure 4b,c. The inconsistent *n*_m_ for BBA produced from the same fuel in repeated experiments (peat and leaf) is indicated by circles of the same color in Figure 4b,c. This supports the substantial variability in the production of INPs through biomass burning, despite aerosol produced from same fuel exhibiting similar chemical compositions. The highest *n*_m_ was observed for fresh hardwood burning aerosol (Figure 4b, red). Apart from this, no significant correlations between fuel types and *n*_m_ of fresh (Figure 4b) or aged BBA (Figure 4c) were identified when considering the variation among repeated experiments from the same fuel type, despite the distinct chemical signatures observed for the different fuels (discussed in Section 3.1). For instance, *n*_m_ of BBA produced from flaming combustion of softwood falls within the *n*_m_ range of BBAs originating from other biomass types and conditions, even though it contains 4 to 400 times higher BC mass concentration than other BBAs (39.2 μg m^–3^ versus 0.01–10.5 μg m^–3^, Table S3). This result confirms the negligible influence of BC on immersion freezing of droplets, aligning with previous studies reporting the poor INAs of BC under mixed-phase cloud conditions.^39,40^ In addition, no correlations (Table S6, the determination of coefficient *R*^2^ < 0.26) were found between the aerosol chemical parameters (O/C, f44 intensity and Org mass fraction) and *n*_m_ at −18 °C or the median freezing temperature (*T*_50_) of BBA. This indicates that the INA of BBA is not sensitive to the chemical parameters used to quantify the composition of the carbonaceous aerosol. This aligns with the findings of Chou et al.,^17^ who demonstrated that the OC/BC did not play an important role in the INA of wood-burning particles. We conclude that carbonaceous materials are not the determining factor for the INA of BBA. Instead, as suggested by other studies, inorganic components, such as ash and minerals in BBA, are likely to serve as INPs.^14,16^ For example, Jahn et al.^16^ reported grass-burning aerosol^6,16^ enriched with mineral-phase exhibit significant INAs at temperatures ranging from −35 to −15 °C. In contrast, they observed wood-burning aerosol showed no INA under the same temperature conditions.^16^ The analysis of the mineral phase was not possible in our study. The distinguished K content detected in BBA produced from different biomass types and burning processes by STXM suggests that the mineral phase content among BBAs may also vary. This variation in mineral phase content of BBA, contributed by the ash particles, may help explain the different INAs of BBA observed in this study. Therefore, we suggest that the presence of mineral-phase in BBA plumes from different combustion sources as well as their INA needs further investigation in airborne atmospheric samples.

**Figure 5:**
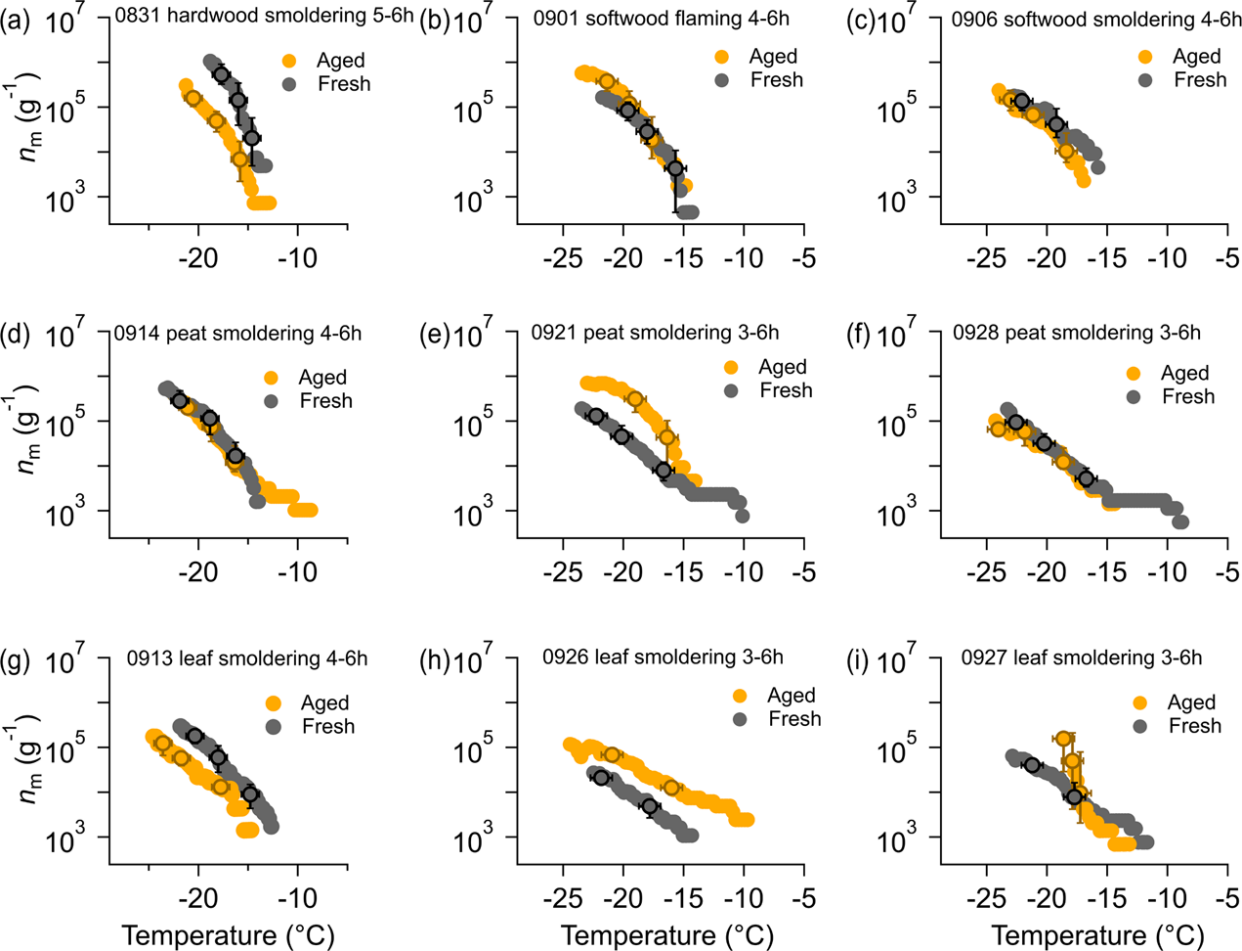
*n*_m_ of fresh (gray circles) and aged (yellow circles) BBA generated at each burning experiment. The horizontal error bars represent a temperature uncertainty of ±0.9 °C in DRINCZ measurements^23^ while the vertical error bars are deviations of *n*_m_ resulting from this temperature uncertainty. Only error bars of a few data points are shown in each panel to avoid overcrowding the figures.

Figure 5 compares the *n*_m_ of fresh and aged BBA generated in each burning experiment. INAs of BBA produced in 4 out of 9 burning experiments (Figure 5b–d,f) show no significant change after a few hours of photochemical aging. An increased *n*_m_ of BBA after aging was observed in 3 burning experiments (Figure 5e,h,i), while *n*_m_ of BBA from 2 burning experiments exhibits up to an order-of-magnitude decrease after aging. The inconsistent change due to aging indicates that the influence of photooxidation on the INA is complex, which is also indicated by previous studies. For example, Jahl et al.^18^ observed an increase in INA on photochemical aging for BBA generated from grass fuels. They attributed this increase to the removal of organic coatings and the exposure of mineral-based ice-active sites upon oxidation. Conversely, Chou et al.^17^ found no observable change in the INAs of wood-burning particles after aging, despite the increase of OC/BC. They explained this by nonuniform organic coatings on particles, where some active sites on the particles remained uncoated and thus no change in INA of these specific sites during aging. Similar to the results from Chou et al.,^17^ we found no correlations between INA of aged BBA and any chemical parameters related to carbonaceous materials (Table S6). The collective results from these three studies imply that the varied influence of photooxidation on the INAs of BBA cannot be explained by changes in the mass and oxygenation of carbonaceous aerosols, but is more likely dependent on the interaction between organic coatings and ice-active particle surfaces, that may be provided by mineral-containing particles in BBAs.^16,18^ This interaction can vary significantly in different burning scenarios. To gain a better understanding of the changing INA in different BBA scenarios during its photochemical aging and the role of organics in this process, further investigations on the progression of inorganic particle surfaces modified by organics during photochemical aging should be conducted. It is also important to note that, after compiling INA results for all types of BBA, no significant change of *n*_m_ has been observed in BBA after aging (Figure S6). This suggests that, under our laboratory photochemical aging conditions, the overall impact of aging on the INAs of BBA appears to be negligible, despite its substantial chemical transformations (discussed in Section 3.2). When considering BBA in the real atmosphere, it represents a complex mixture of aerosols originating from diverse combustion sources. Thus, if we assume aging BBA produced from same biomass types and under similar radiation conditions as used in our laboratory measurements, no notable changes in INAs of produced BBA would be expected.

### 3.3 Atmospheric Implications and Conclusions

**Figure 6:**
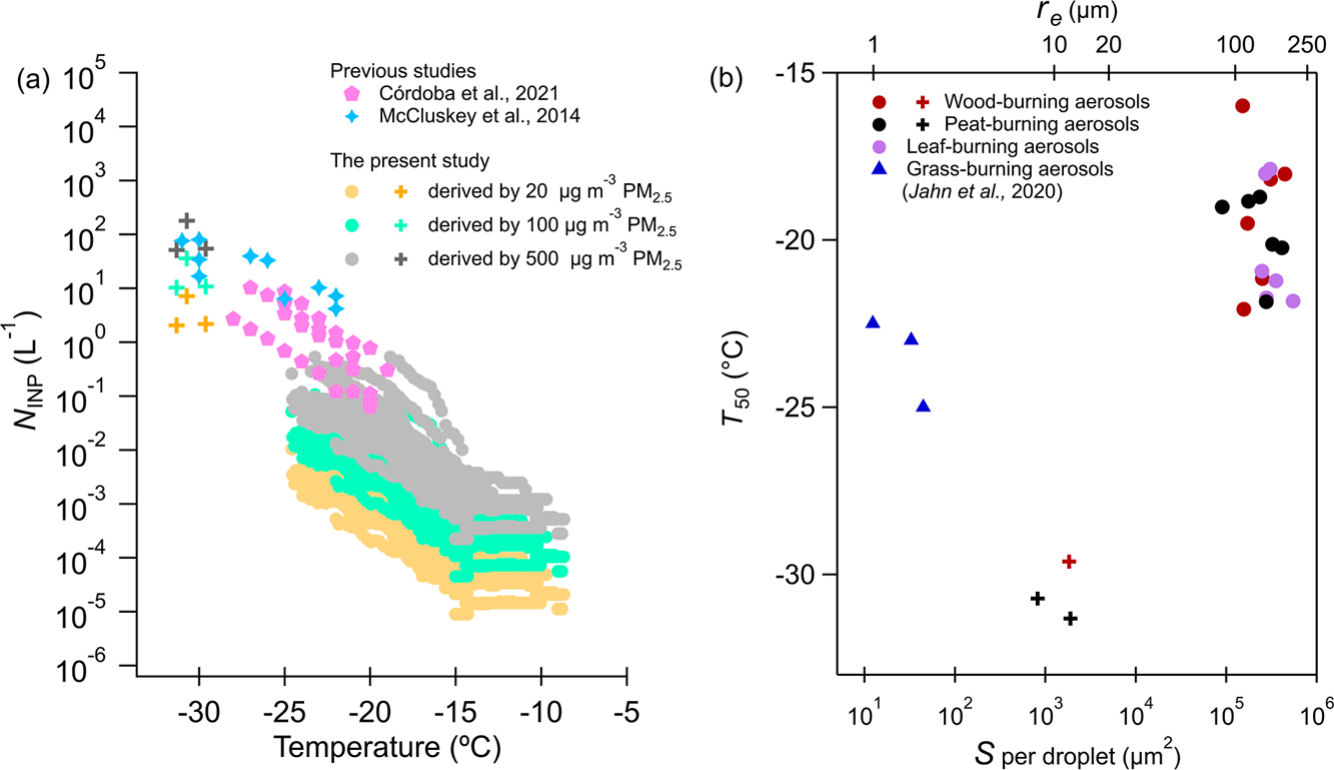
Parameters to assess the atmospheric relevance of INPs produced by laboratory-controlled burns. (a) INP concentration per liter air (*N*_INP_) as a function of temperature; (b) median freezing temperature (*T*_50_) as a function of equivalent INP surface area per droplet (*S*, bottom axis) and the equivalent aerosol particle radius needed in one cloud droplet (*r*_e_, top axis). *N*_INP_ is calculated based on eq 4, using the detected *n*_m_ and assuming the PM_2.5_ mass concentrations of 20, 100, and 500 μg m^–3^, as those obtained in McCluskey et al.^12^ and Córdoba et al.^41^ during the BBA polluted period. *N*_INP_ values reported by McCluskey et al.^12^ and Córdoba et al.^41^ are also presented in (a) for comparison. *S* and *r*_e_ are derived according to eq 5, assuming a spherical particle with a density of 1.26 g cm^–3^. *S* and *r*_e_ of grass-burning particles are also shown in (b) for comparison (blue triangles), which was derived based on *n*_m_ values reported by Jahn et al.^16^ Data from DRINCZ and MINCZ are denoted by circles and crosses, respectively.

The atmospheric relevance of our results can be assessed by calculating the INP concentration (*N*_INP_) that could be contributed by atmospheric BBA plumes. *N*_INP_ is derived based on eq 4, using the *n*_m_ and a PM_2.5_ mass concentration in a BBA plume reported by McCluskey et al.^12^ and Córdoba et al.^41^ during realistic prescribed and wildfire events. Three values of PM_2.5_ mass concentration are assumed (20, 100, 500 μg m^–3^), reflecting the range observed during these BBA events (20–500 μg m^–3^).^12,41^ The resulting *N*_INP_ values derived using the *n*_*m*_ from this study and PM_2.5_ from McCluskey et al.^12^ and Córdoba et al.^41^ are shown in Figure 6a and compared to the *N*_INP_ values measured in those studies. The estimated *N*_INP_ is 1–3 orders of magnitude lower than the measured values, indicating our laboratory-controlled burns produce an overall lower INP concentration than those measured in atmospheric BBA plumes. The higher INP concentrations observed for BBA plumes in field measurements may be explained by the mixing of the BBA plumes with other INPs, such as the colofted soil dust and ash^8,14^ or other background INPs (e.g., biological particles). In addition, if fuels that efficiently produce ice-active mineral-containing particles, such as grass,^6,16^ also burn during these events, they can generate more INPs compared to the biomasses investigated here. The two data sets become closer when a higher PM_2.5_ mass concentration (e.g., 500 μg m^–3^) was assumed, indicating that BBA produced from laboratory-controlled burns will only have considerable influence on the atmospheric INP population in environments significantly perturbed by such emissions.

Figure 6b shows *T*_50_ of droplets in relation to the spherical equivalent INP radius (*r*_e_, upper axis) and the corresponding total surface area (*S*, bottom axis) required per cloud droplet to initiate immersion freezing. Details on the derivation of *r*_e_ and *S* can be found in Section 2.2. Based on the calculation, INPs produced from laboratory-controlled burns here would need to reach a radius of 100–200 μm to efficiently nucleate ice at temperatures above −22 °C (Figure 6b). Needless to say, individual biomass burning particles of such size are likely absent in the real atmosphere. As shown in Figure 3 (IMG 1–5), particles collected on TEM grids can only reach a maximum size of a few micrometers. Hu et al.^27^ reported that wood-burning particles produced from a similar procedure have a size smaller than 500 nm. In other words, DRINCZ has detected moderate INA of BBA produced here because the total particle mass per droplet is much larger than the mass of particles of a few micrometers. These particles will not be ice-active under atmospheric-relevant particle size conditions. This also explains the absence of immersion freezing of droplets with picoliter size in MINCZ measurements, as the droplets are much smaller than those in DRINCZ measurements and thus with much smaller BBA mass in them (Figure S2). For a BB particle to serve as immersion-freezing INP at temperatures relevant to MINCZ measurements (<−30 °C), it must reach a radius of 8–10 μm (Figure 6b, crosses), a particle size that is also rare in the atmosphere. The *r*_e_ of grass-burning aerosols enriched with minerals is also derived, based on *n*_m_ reported by Jahn et al.^16^ (blue triangles in Figure 6b). These mineral-rich BB particles can nucleate ice at temperatures ranging from −22.3 to −25 °C with a radius of 1–2 μm, which we consider to be more atmospherically relevant and probable. Particles of such size are common in biomass burning events, from ash and lofted soil particles.^42,43^ This comparison again implies that the limited INA of BBA obtained in our study may be attributed to low mineral-phase content.

## Supporting Information Available

The results of sample and background droplet freezing experiments (Figures S1 and S2, Table S7, Text S3); details about the chamber operations (Tables S1 and S2, Texts S1 and S2); chemical characterizations of BBA (Tables S3–S6, Figures S3–S5); ice nucleation activities of BBA (Table S6, Figure S6) (PDF)
