# Supplementary material for: Ice Nucleation Abilities and Chemical Characteristics of Laboratory-Generated and Aged Biomass Burning Aerosols
Source: Environ Sci Technol. 2025 Feb 2;59(5):2575–86. doi: 10.1021/acs.est.4c04941 (PMC11823448; doi:10.1021/acs.est.4c04941)
Supplement: Supplementary file 1 — es4c04941_si_001.pdf [file es4c04941_si_001.pdf]

Supporting Information for

Ice Nucleation Abilities and Chemical Characteristics of Laboratory-Generated and Aged Biomass Burning Aerosols

Jie Chen[1,*], Fähndrich Martin Othmar Jakob[1], Aristeidis Voliotis[2,4], Huihui Wu[2], Sara Aisyah Syafira[2], Osayomwanbor Oghamaa[2], Nadia Shardt[1], Nicolas Fauré[3], Xiangrui Kong[3], Gordon Mcfiggans[2], Zamin A. Kanji[1,*]

1 Institute for Atmospheric and Climate Science, ETH Zürich, Zurich, 8092, Switzerland

2 Centre for Atmospheric Science, Department of Earth and Environmental Sciences, School of Natural Sciences, University of Manchester, Manchester, M13 9PL, UK

3 Department of Chemistry and Molecular Biology, University of Gothenburg, SE-413 90 Gothenburg, Sweden

4 National Centre for Atmospheric Science, The University of Manchester, M13 9PL, UK

Correspondence to: jie.chen@env.ethz.ch and zamin.kanji@env.ethz.ch

Summary: 13 pages, 6 figures, 7 tables

Table of contents:

Table S1. Sampling information on collected biomass burning samples....................................S3.

Table S2. Timeline of chemical composition measurements of bulk BBA..................................S4.

Table S3. Mass concentrations of chemical components in fresh and aged BBA.........................S5.

Table S4. The transition energy of functional groups detected in individual biomass burning particles by STXM-NEXAFS...S6.

Table S5. The ratio of absorption at 285.0 eV to 305.0 eV (285/305 eV) for particles produced in each experiment...S6.

Table S6. Correlations between determined aerosol chemical parameters and n_m of BBA at -18 °C or T_{50} of sample droplets...S6.

Table S7. Fitting curve parameters of the frozen fraction ($\mathrm{f_{ice}}$) of droplets obtained from blank filter suspensions...S9.

Figure S1. Frozen fraction ($\mathrm{f_{ice}}$) of biomass burning aerosol (BBA) sample droplets and filter background droplets measured by DRINCZ (a) and MINCZ (b)..S8.

Figure S2. Freezing temperatures of BBA sample droplets and filter background droplets measured by MINCZ...S9.

Figure S3. Chemical compositions of fresh BBA generated in each experiment........................S10.

Figure S4. Chemical compositions of aged BBA generated in each experiment........................S11.

Figure S5. Carbon K-edge spectra detected at different areas within the individual particles.S12.

Figure S6. Active site density per unit of particle mass (n_m) of fresh (gray circles) and aged (yellow circles) BBA...S13.

Text S1. Control of combustion type...S7.

Text S2. Hash cleaning procedure of Manchester Aerosol Chamber..S7.

Text S3. Calculation and exclusion of filter background...S7.

Table S1. Sampling information on biomass burning samples collected from chamber experiments.

Date	Biomass type	Burning conditions	Fresh/Aged	Particle Mass (g)	Filter samples			Grid samples		
					Sampling time	Flow rate (L min^{-1})	Aging time (h)	Sampling time	Flow rate (L min^{-1})	Aging time (h)
31/08	Hardwood	Smoldering	fresh	0.0006	5 min	3.5	5–6	5 min	1	5–6
			aged	0.0020	1 h	3.5		1 h	1	
01/09	Softwood	Flaming	fresh	0.0061	5 min	3.5	4–6	5 min	1	5.5–6
			aged	0.0008	2 h	3.5		1 h	1	
06/09	Softwood	Smoldering	fresh	0.0003	5 min	3.5	4–6	5 min	1	5.5–6
			aged	0.0012	2 h	3.5		1 h	1	
13/09	Leaf	Smoldering	fresh	0.0017	5 min	3.5	4–6	5 min	1	5–6
			aged	0.0010	2 h	3.5		1 h	1	
27/09	Leaf	Smoldering	fresh	0.0019	5 min	3.5	3–6	5 min	1	5–6
			aged	0.0021	3 h	3.5		1 h	1	
14/09	Peat	Smoldering	fresh	0.0009	5 min	3.5	4–6	5 min	1	5–6
			aged	0.0014	2 h	3.5		1 h	1	
21/09	Peat	Smoldering	fresh	0.0019	5 min	3.5	3–6	5 min	1	4.5–5.5
			aged	0.0003	3 h	3.5		1 h	1	
28/09	Peat	Smoldering	fresh	0.0026	5 min	3.5	3–6	5 min	1	5–6
			aged	0.0010	3h	3.5		1 h	1	

Table S2. Timeline of chemical composition measurements of bulk BBA by C-TOF-AMS and SP2.

**Date**	Aerosol injection	Light on	Aged filter sample start	Aged filter sample end	Light off
31/08	10:22:00	11:03:00	16:20:00	17:25:00	17:25:00
01/09	10:11:00	10:44:00	14:45:00	16:50:00	16:50:00
06/09	10:14:00	11:04:00	14:50:00	17:06:00	17:06:00
13/09	09:47:00	10:49:00	14:45:00	17:00:00	17:00:00
14/09	10:01:00	10:49:00	14:46:00	16:58:00	16:58:00
21/09	10:05:00	10:47:00	13:50:00	16:52:00	16:52:00
26/09	09:56:00	10:40:00	13:40:00	16:45:00	16:45:00
27/09	09:59:00	10:36:00	13:37:00	16:38:00	16:38:00
28/09	09:46:00	10:30:00	13:20:00	16:32:00	16:32:00

C-TOF-AMS: The compact time-of-flight aerosol mass spectrometer;

SP2: single particle soot photometer;

Table S3. Mass concentrations* of chemical components in fresh and aged BBA generated in each experiment.

	08/31	09/01	09/06	09/13	09/27	09/14	09/21	09/28
	Hardwood smoldering	Softwood flaming	Softwood smoldering	Leaf smoldering	Leaf smoldering	Peat smoldering	Peat smoldering	Peat smoldering
Fresh BBA (μg m^{-3})								
Org	40.79±6.56	4.47±0.41	28.70±4.21	79.60±6.27	62.02±3.43	62.33±12.26	113.97±12.51	96.37±15.92
NO_3^-	2.86±0.20	0.46±0.01	1.66±0.15	2.44±0.10	2.24±0.07	0.49±0.08	0.82±0.06	0.78±0.10
NH_4^+	0.34±0.08	0.01±0.01	0.25±0.02	0.07±0.02	0.10±0.01	0.04±0.03	0.02±0.02	0.26±0.06
SO_4^{2-}	0.36±0.06	0.06±0.01	0.14±0.02	0.21±0.03	0.24±0.03	0.25±0.04	0.41±0.04	0.45±0.06
Chl^-	0.25±0.04	0.06±0.01	0.19±0.05	0.32±0.04	0.39±0.04	0.17±0.03	0.27±0.03	0.37±0.05
BC	10.5±0.96	39.2±2.77	6.84±0.70	**0.45**±0.06	**1.74**±0.14	**0.02**±0.01	**0.01**±0.00	**0.02**±0.00
BC/PM	19.0%±1%	88.6%±0.2%	18.1%±0.7%	0.5%±0.0%	2.6%±0.1%	0.0%±0.0%	0.0%±0.0%	0.0%±0.0%
Org/PM	74.1%±1.5%	10.1%±0.2%	76.0%±0.9%	95.8%±0.13%	93.0%±0.1%	98.5%±0.0%	98.7%±0.0%	98.1%±0.0%
Org/BC	3.90±0.27	0.11±0.00	4.20±0.20	-	-	-	-	-
O/C	0.35±0.01	0.40±0.01	0.33±0.00	0.26±0.01	0.27±0.01	0.17±0.00	0.15±0.00	0.16±0.00
Aged BBA (μg m^{-3})								
Org	2.99±0.68	0.87±0.29	2.25±1.02	3.82±2.26	3.57±2.63	1.82±0.93	7.43±4.65	3.84±2.92
NO_3^-	0.16±0.04	0.07±0.02	0.13±0.05	0.19±0.10	0.21±0.15	0.04±0.02	0.12±0.05	0.10±0.06
NH_4^+	0.05±0.01	0.05±0.01	0.02±0.01	0.01±0.01	0.02±0.01	-	-	0.02±0.01
SO_4^{2-}	0.12±0.01	0.23±0.03	0.07±0.00	0.06±0.01	0.03±0.01	0.06±0.01	0.10±0.01	0.06±0.02
Chl^-	0.01±0.00	0.00±0.00	0.01±0.00	0.01±0.01	0.01±0.01	-	0.02±0.01	0.01±0.01
BC	0.49±0.11	2.68±1.27	0.45±0.20	**0.06**±0.01	**0.14**±0.09	**0.02**±0.00	**0.03**±0.01	**0.03**±0.01
BC/PM	12.9%±0.4%	68.7%±5.4%	15.5%±0.9%	1.5%±0.8%	3.4%±0.6%	1.2%±0.8%	0.4%±0.3%	0.8%±0.7%
Org/PM	78.1%±0.9%	22.4%±1.5%	76.9%±1.8%	92.0%±1.6%	89.8%±1.0%	93.1%±2.4%	96.4%±1.3%	94.5%±2.1%
Org/BC	6.04±0.23	0.33±0.05	4.97±0.35	-	-	-	-	-
O/C	0.73±0.01	0.74±0.05	0.64±0.04	0.45±0.04	0.47±0.04	0.34±0.05	0.26±0.03	0.32±0.04

*The reported mass concentration of chemical components in BBA is the average value±standard deviation recorded by C-TOF-AMS during the filter sampling time. Fresh BBA: averaged data recorded from "Aerosol injection" to "Light on", as indicated in Table S2; Aged BBA: averaged data recorded from "Aged sample start" to "Aged sample end", as indicated in Table S2.

Table S4. The transition energy of functional groups detected in individual biomass burning particles by STXM-NEXAFS (synchrotron-based scanning transmission X-ray microscopy (STXM) coupled with near-edge X-ray absorption fine structure spectroscopy). All information can be found in the overview paper from Moffet *et al.* [1].

Transition energy (eV)	Functional groups
285	C 1s→ $\pi^*_{R(C^*=C)R}$
286.8	C 1s → $\pi^*_{R(C^*=O)R}$<br>C 1s → $\pi^*_{quinone(C^*=O)}$<br>C 1s → $\pi^*_{phenolic(C^*–OH)}$
288.4	C 1s → $\pi^*_{R(C^*=O)OH}$
292.5	C 1s → $\sigma^*_{C^*–C}$
297.1	K $2p_{1/2}$→ σ^*
299.6	K $2p_{3/2}$→σ^*

Table S5. The ratio of absorption at 285.0 eV to 305.0 eV (285/305 eV) for particles produced in each experiment.

Biomass type	ratio
leaf smoldering	0.57
peat smoldering	0.69
hardwood smoldering	0.79
softwood smoldering	0.92
softwood flaming	0.97

Table S6. Correlations between determined aerosol chemical parameters (O/C, f44 intensity, Org/BC, Org mass fraction) and n_m of BBA at -18 °C or median freezing temperature (T_{50}) of sample droplets. The goodness of the correlations is indicated by the coefficient of determination (R2).

R^2 of Fresh BBA		
Aerosol chemical parameters	n_m at -18 °C	T_{50}
O/C	0.14	0.26
f44	0.14	0.26
Org mass fraction	0.01	0.11
R^2 of Aged BBA		
Aerosol chemical parameters	n_m at -18 °C	T_{50}
O/C	0.19	0.02
f44	0.19	0.02
Org mass fraction	0.07	0.01

Text S1. Control of combustion type. The flaming phase burning is defined as combustion occurring with the stove ventilation fully open where an adequate oxygen supply is ensured, and with the visual presence of flames during burning. In contrast, the smoldering burning phase refers to burns conducted with the air ventilation turned off and the absence of flame.

Text S2. Hash cleaning procedure of Manchester Aerosol Chamber. Harsh cleaning is a more aggressive cleaning procedure for the MAC chamber. In this procedure, particle-free compressed air and high levels of ozone (>1 ppm) and water vapor (RH>70%) are added to the chamber with illumination, where the atmosphere of the chamber undergoes several hours of photooxidation. This procedure is carried out weekly during an experimental campaign and is sufficient to ensure a low level of particle and gas pollutant backgrounds in the chamber, as indicated by our chamber validation experiments in Shao *et al.* [2].

Text S3. Calculation and exclusion of filter background. The frozen fraction of the droplets derived from blank filters ($f_{\mathrm{ice,BG}}$) is shown in Fig. S1, with blue circles in (a) obtained from DRINCZ and (b) for MINCZ measurements. The temperature dependence of $f_{\mathrm{ice,BG}}$ is described by the sigmoid fitting curve with a 95% confidence interval (blue shading) in Fig. S1 (calculated using the built-in Python package [curve_fit]). The fitting of the measured data points has included the temperature uncertainty for DRINCZ (0.9 °C) and MINCZ measurements (0.2 °C). The root mean square error (RMSE) of $f_{\mathrm{ice,BG}}$ is 0.05 and 0.18 for DRINCZ and MINCZ measurements, respectively, indicating a sufficiently good fit. The functions of the fitting curves and their lower and upper limits are provided in Table S7. It is important to note that the function of the upper limit curve is used to derive the number concentration of ice nucleating particles (INPs) per unit volume of droplet ($K(T)_{BG}$) based on Eq. (2) in the main text. The $K(T)_{BG}$ is then subtracted from $K(T)$ of BBA samples (Eq. (3)), to determine the background corrected INP concentrations for BBA, as explained in the main text.

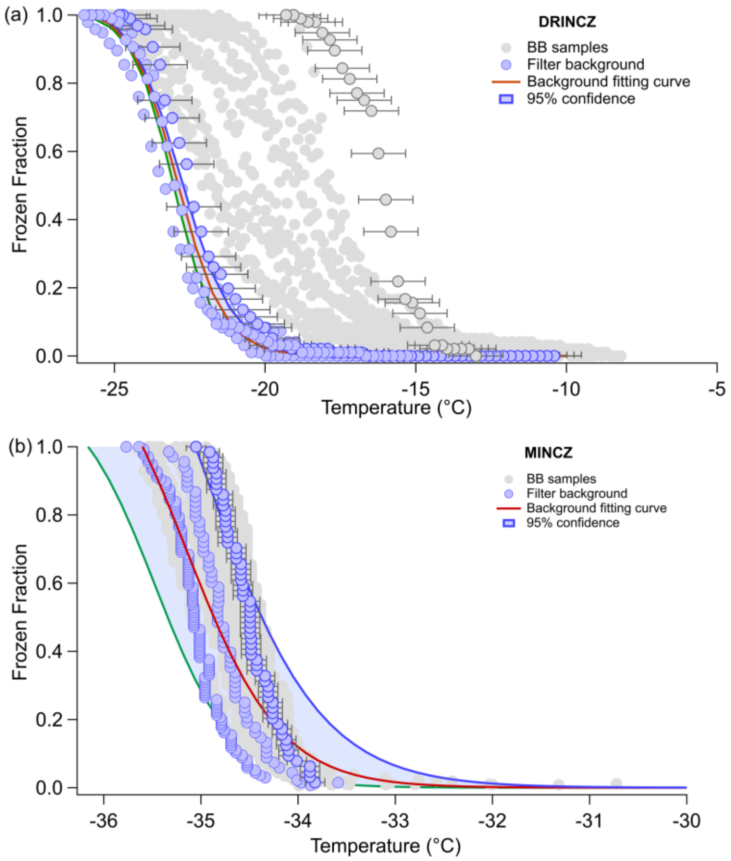

Figure S1. Frozen fraction (f_{ice}) of biomass burning aerosol (BBA) sample droplets and filter background droplets measured by DRINCZ (a) and MINCZ (b). The red lines are the fitting curves of f_{ice} of background (particle-free) droplets. The blue shading represents the 95% confidence interval of the fit. The green lines and blue lines are the lower limit and upper limit of this interval respectively. The horizontal error bars represent the instrument temperature uncertainty, which is ±0.9 °C for DRINCZ and ±0.2 °C for MINCZ, as indicated in David *et al.* [3] and Isenrich *et al.* [4]. Only error bars of select data points are shown here for clarity.

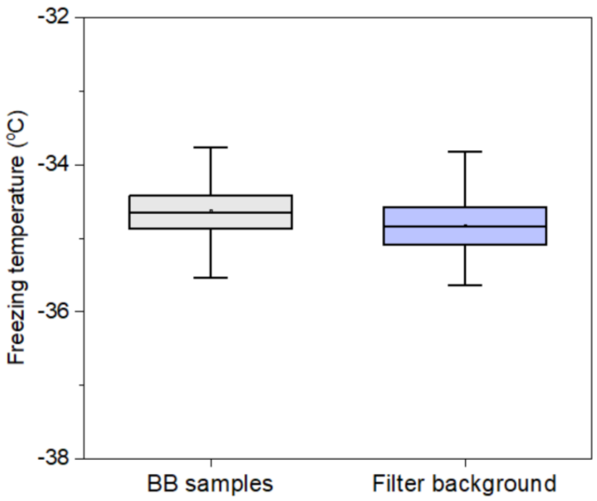

Figure S2. Freezing temperatures of biomass burning aerosol (BBA) sample droplets (gray box) and filter background droplets (blue box) measured by MINCZ. The lower, middle and upper edges of the box represent the first quartile (25th percentile), median (50th percentile) and the third quartile (75th percentile) of dataset, respectively.

Table S7. Fitting curve parameters of the frozen fraction (f_{ice}) of droplets obtained from blank filter suspensions for DRINCZ and MINCZ measurements. The upper and lower limits of the fitting results are from the 95% confidence interval.

$y = L/(1 + \exp[-k(x - x_0)])$			
DRINCZ			
	L	k	x_0
Upper limit	1.051	-1.145	-22.808
Fitting curve	1.028	-1.288	-22.920
Lower limit	1.010	-1.459	-23.047
MINCZ			
	L	k	x_0
Upper limit	1.858	-1.692	-34.966
Fitting curve	1.391	-2.069	-35.146
Lower limit	1.186	-2.446	-35.471

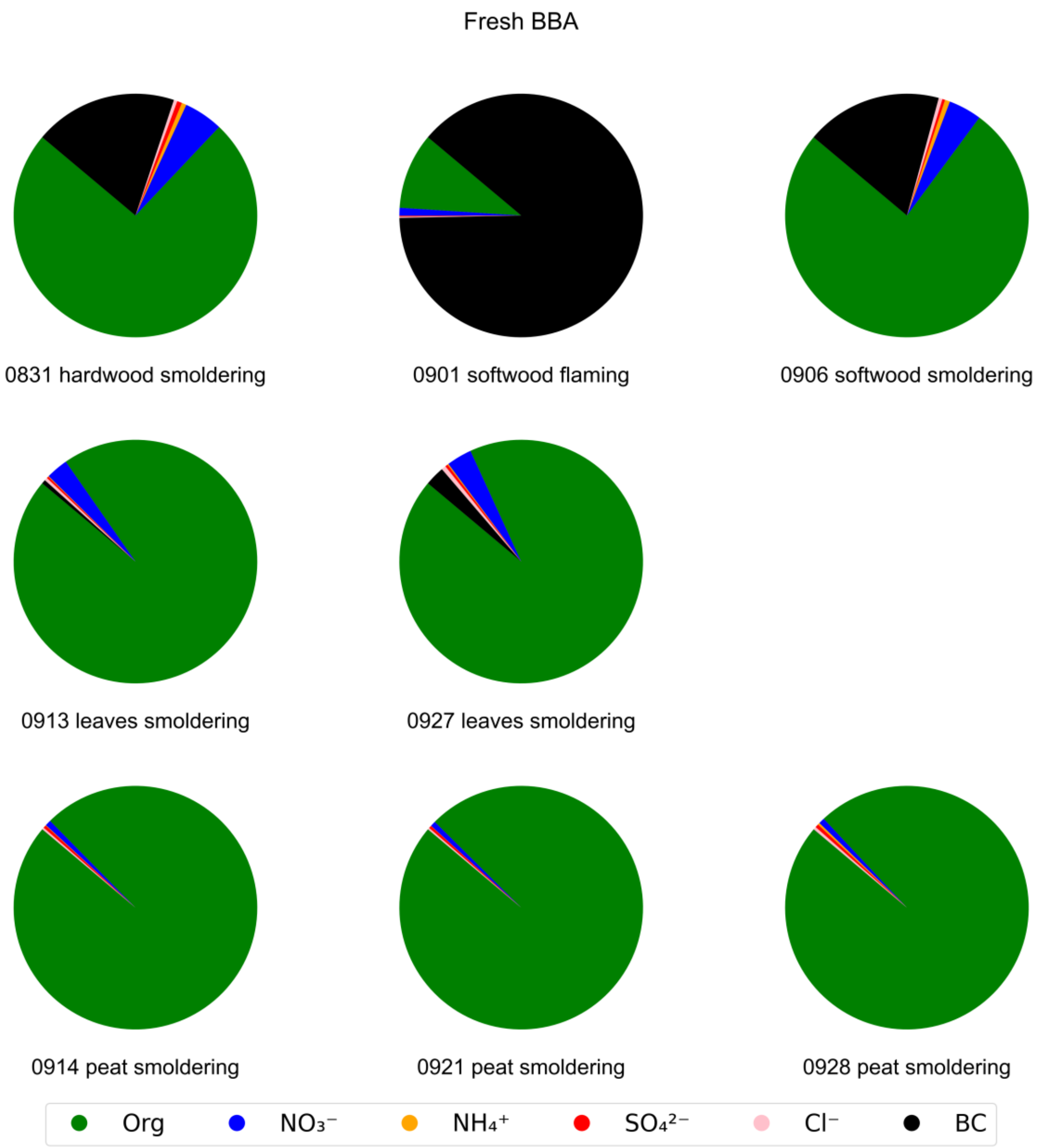

Figure S3. Chemical compositions of fresh BBA generated in each experiment. The organic compounds (Org), sulfate (SO_4^{2-}), nitrate (NO_3^-), ammonium (NH_4^+) and chloride (Cl^-) were measured by C-ToF-AMS (high-resolution time-of-flight aerosol mass spectrometer) and the black carbon (BC) were measured by SP2 (single particle soot photometer). Note that the mass fraction of each chemical component represents the mean value measured over time (~30 to 60 minutes) before photochemical oxidation (from "Aerosol injection" to "Light on", as given in Table S2).

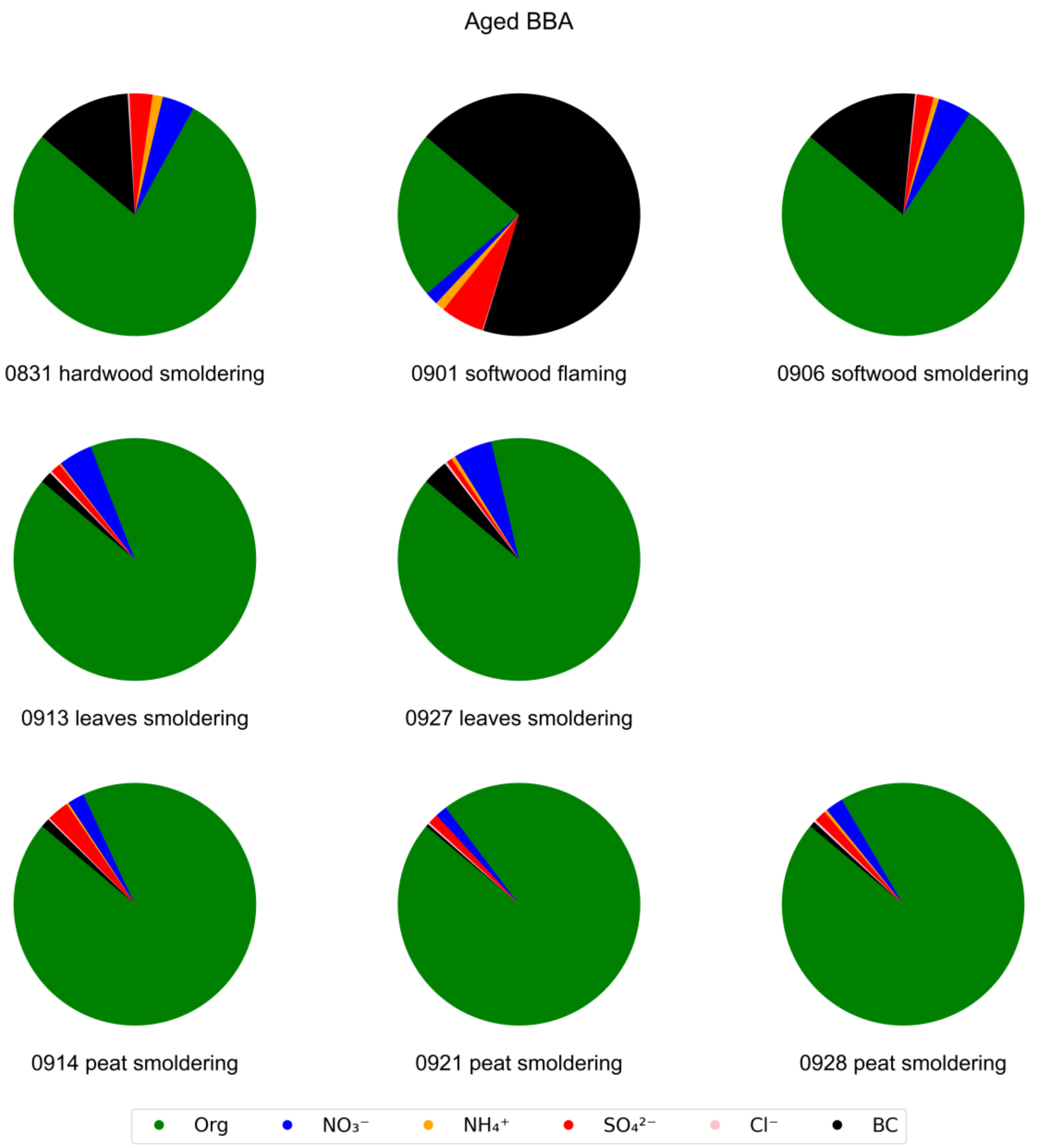

Figure S4. Chemical compositions of aged BBA generated in each experiment. The organic compounds (Org), sulfate (SO_4^{2-}), nitrate (NO_3^-), ammonium (NH_4^+) and chloride (Cl^-) were measured by C-ToF-AMS (high-resolution time-of-flight aerosol mass spectrometer) and the black carbon (BC) were measured by SP2 (single particle soot photometer). Note that the mass fraction of each chemical component represents the mean value measured over time (~1~3 hours, Table S1) during photochemical oxidation. The total measuring time of aged BBA by C-TOF-AMS and SP2 in each experiment is the same as the collecting time of the aged filter samples (from “Aged filter sample start” to “Aged filter sample end”, as given in Table S2).

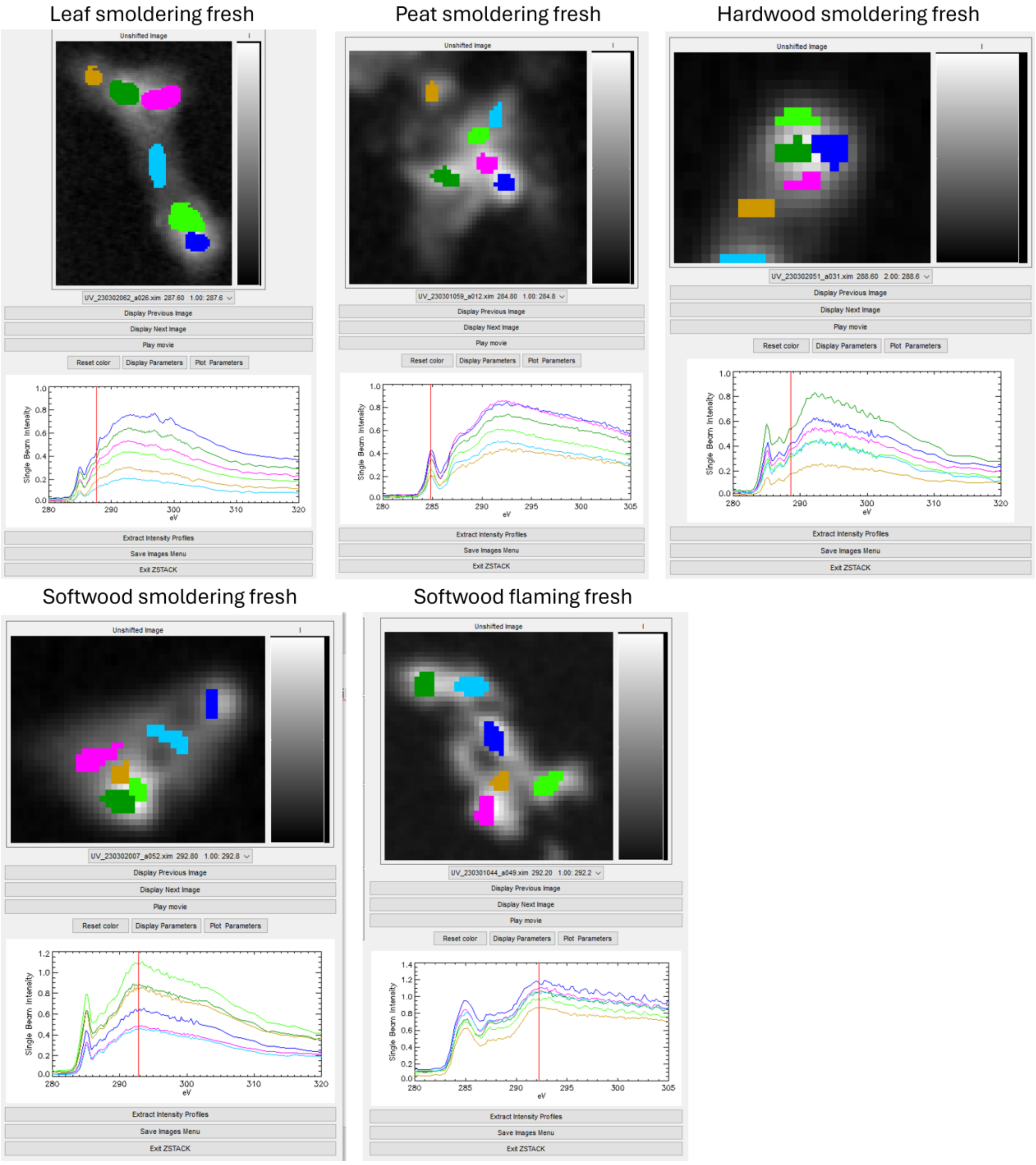

Figure S5. Carbon K-edge spectra detected at different areas within the individual particles. Each spectrum shown in the lower panel of the figure corresponds to the composition obtained from the area of the same color within the particle (upper panel of each figure). The similar features observed in spectra obtained from different areas of the particle indicate the homogeneity of carbon composition within the detected particle.

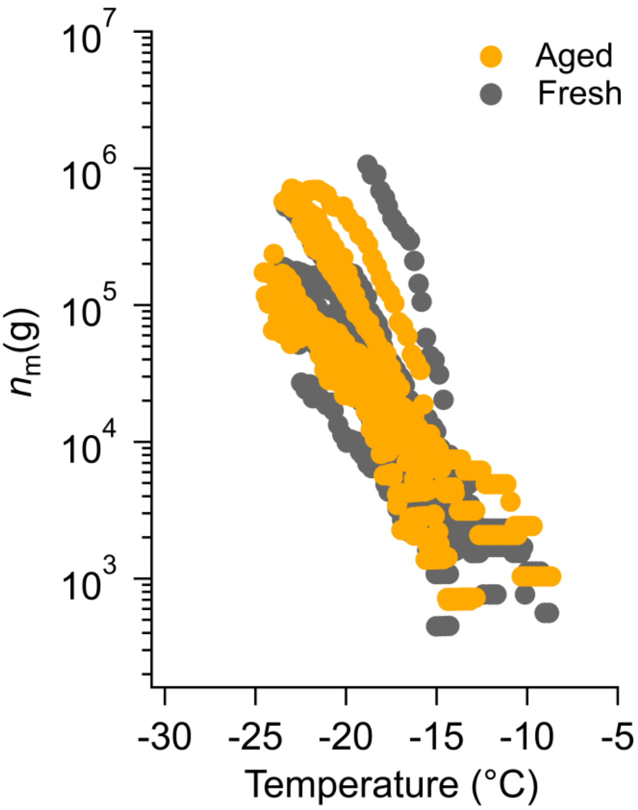

Figure S6. Active site density per unit of particle mass (n_m) of fresh (gray circles) and aged (yellow circles) BBA as a function of temperature generated from different fuels and burning conditions.

References

(1) Moffet, R. C.; Tivanski, A. V.; Gilles, M. K. *Scanning transmission X-ray microscopy: Applications in atmospheric aerosol research*; Lawrence Berkeley National Lab.(LBNL), Berkeley, CA (United States), 2011. DOI: 10.1201/b10417-22.

(2) Shao, Y.; Wang, Y.; Du, M.; Voliotis, A.; Alfarra, M. R.; O'Meara, S. P.; Turner, S. F.; McFiggans, G. Characterisation of the Manchester Aerosol Chamber facility. *Atmos. Meas. Tech.* **2022**, *15* (2), 539-559. DOI: 10.5194/amt-15-539-2022.

(3) David, R. O.; Cascajo-Castresana, M.; Brennan, K. P.; Rösch, M.; Els, N.; Werz, J.; Weichlinger, V.; Boynton, L. S.; Bogler, S.; Borduas-Dedekind, N.; Marcolli, C.; Kanji, Z. A. Development of the DRoplet Ice Nuclei Counter Zurich (DRINCZ): validation and application to field-collected snow samples. *Atmos. Meas. Tech.* **2019**, *12* (12), 6865-6888. DOI: 10.5194/amt-12-6865-2019.

(4) Isenrich, F. N.; Shardt, N.; Rösch, M.; Nette, J.; Stavrakis, S.; Marcolli, C.; Kanji, Z. A.; deMello, A. J.; Lohmann, U. The Microfluidic Ice Nuclei Counter Zürich (MINCZ): a platform for homogeneous and heterogeneous ice nucleation. *Atmos. Meas. Tech.* **2022**, *15* (18), 5367-5381. DOI: 10.5194/amt-15-5367-2022.
